# Supplementary material for: Human Coronavirus 229E Infection Inactivates Pyroptosis Executioner Gasdermin D but Ultimately Leads to Lytic Cell Death Partly Mediated by Gasdermin E
Source: Viruses. 2024 Jun 1;16(6):898. doi: 10.3390/v16060898 (PMC11209299; doi:10.3390/v16060898)
Supplement: Supplementary file 1 [file viruses-16-00898-s001.zip › viruses-2964720-supplementary.pdf]

A

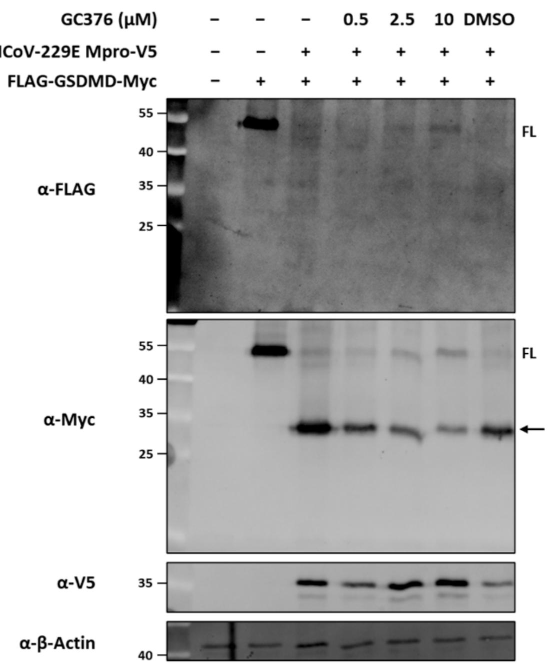

Figure S1. Cleavage of GSDMD by Mpro in the presence of Mpro inhibitor GC376. (A) FLAG-GSDMD-Myc (2 µg/well) and V5-tagged HCoV-229E WT Mpro (2 µg/well) mammalian expression plasmids were co-transfected in HEK293T cells and cells were treated with increasing concentrations of Mpro inhibitior GC376. At 24h post-transfection, cells were lysed for immunoblotting. Immunoblots are representative of two independent experiments. FL, full-length; arrows indicate cleaved fragments identified by immunoblotting.

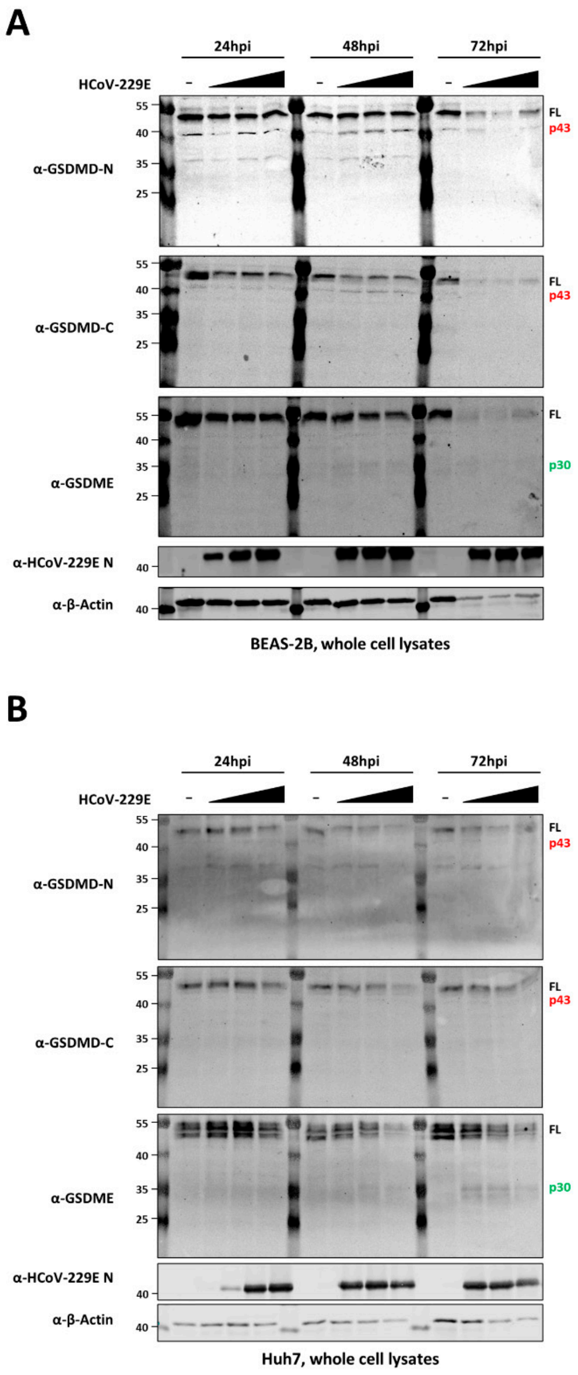

Figure S2. HCoV-229E infection leads to inactivation of GSDMD and activation of GSDME. BEAS-2B (A) and Huh7 (B) cells were mock infected or infected with HCoV-229E at MOIs of 0.01, 1 and 3. Whole cell lysates were harvested at the indicated time points and analysed by immunoblotting for GSDMD N-terminal, GSDMD C-terminal, GSDME, HCoV-229E nucleocapsid protein and β-actin. FL: full-length.

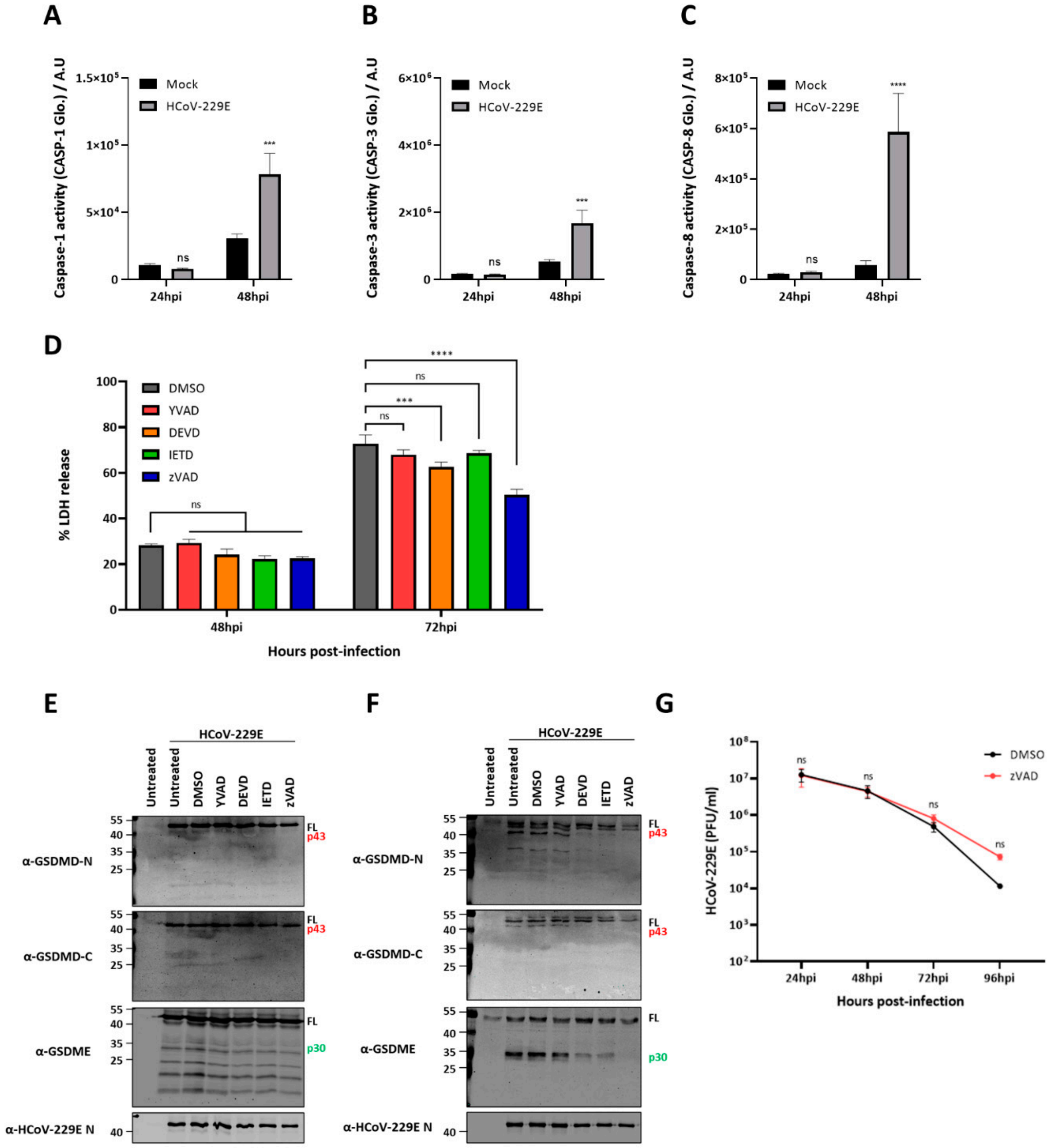

Figure S3. Pan-caspase inhibition dampens virus-induced lytic cell death in Huh7 cells. (A-C) Huh7 cells were mock infected or infected with HCoV-229E MOI 3 and caspase activity was measured at 24 and 48 hpi; (D) Huh7 cells were treated with vehicle (DMSO), 40 μM Ac-YVAD-cmk, 20 μM z-DEVD-fmk, 25 μM z-IETD-fmk or 25 μM z-VAD, and mock infected or infected with HCoV-229E at an MOI of 3, and LDH release was then assessed at the indicated time points; (E and F) at 48 h post-infection, supernatants of HCoV-229E infected BEAS-2B (E) and Huh7 (F) cells treated with vehicle (DMSO), Ac-YVAD-cmk, z-DEVD-fmk, z-IETD-fmk or z-VAD were harvested for immunoblotting analysis; and (G) supernatants of infected Huh7 treated with either vehicle (DMSO) or z-VAD were harvested at the indicated time points, and HCoV-229E infectious virus particles were quantified by plaque assay. Experiments were performed as triplicates and repeated at least trice. A.U, Arbitrary units; LDH, lactate dehydrogenase; MOI, multiplicity of infection; ns, not significant;; ***, $p < 0.0001$; ****, $p < 0.00001$.
